# Supplementary material for: Ubiquitin receptors are required for substrate-mediated activation of the proteasome’s unfolding ability
Source: Sci Rep. 2019 Oct 10;9:14506. doi: 10.1038/s41598-019-50857-y (PMC6787058; doi:10.1038/s41598-019-50857-y)
Supplement: Supplementary file 1 — Supplementary Information [file 41598_2019_50857_MOESM1_ESM.pdf]

# Ubiquitin receptors are required for substrate-mediated activation of the proteasome's unfolding ability

*Mary D. Cundiff, Christina M. Hurley, Jeremy D. Wong, Joseph A. Boscia IV, Aarti Bashyal, Jake Rosenberg, Eden L. Reichard, Nicholas D. Nassif, Jennifer S. Brodbelt & Daniel A. Kraut*

**Supplementary Information**

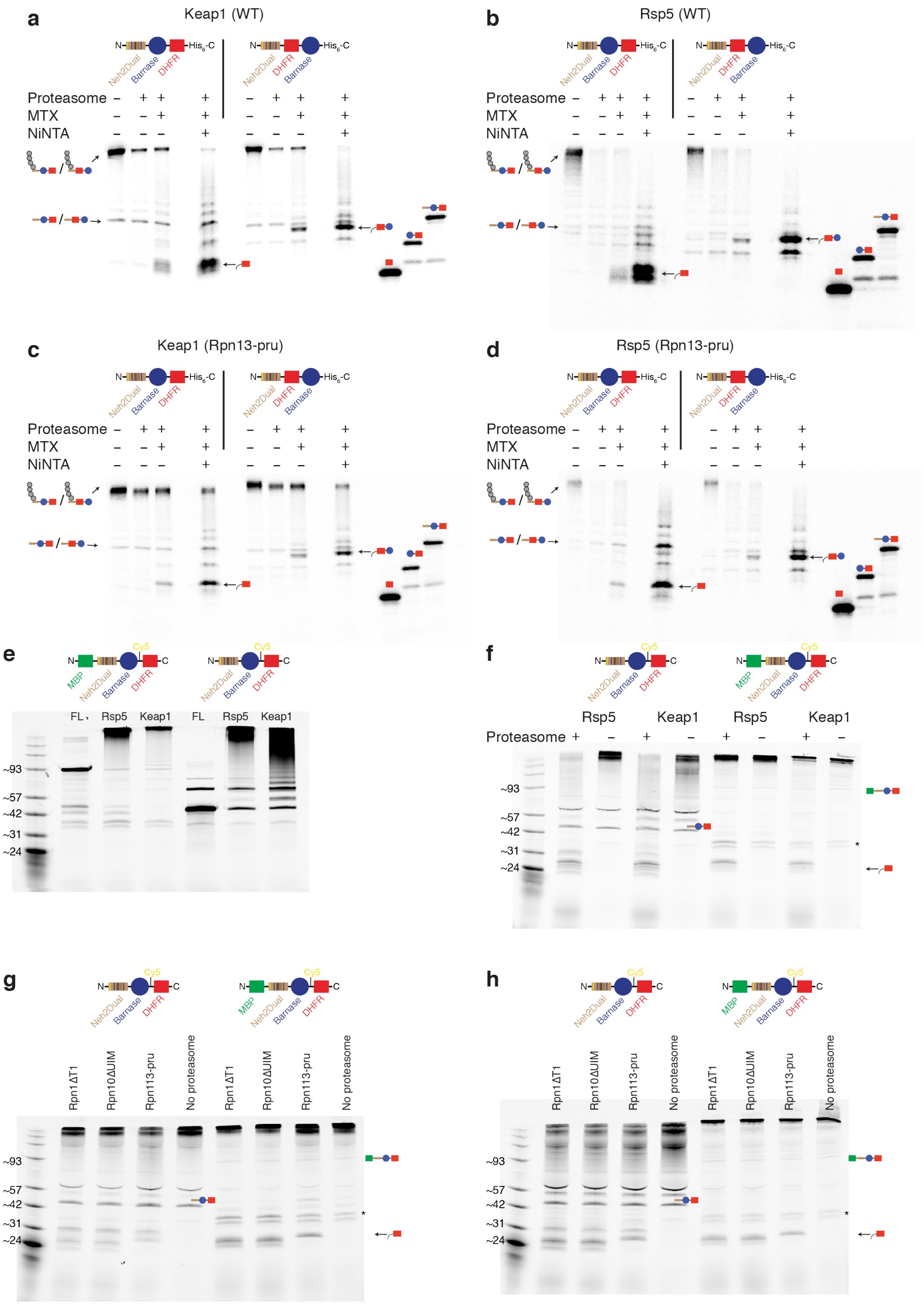
a
Keap1 (WT)
b
Rsp5 (WT)
c
Keap1 (Rpn13-pru)
d
Rsp5 (Rpn13-pru)
N-
His6-C
Neh2Dual
Barnase
DHFR
Proteasome
MTX
NiNTA
e
MBP
Cy5
FL
Rsp5
Keap1
~93
~57
~42
~31
~24
f
Rsp5
Keap1
Proteasome
g
h
Rpn1ΔT1
Rpn10ΔUIM
Rpn13-pru
No proteasome
*

**Supplementary Figure S1**. Degradation proceeds from the N-terminal degron towards the C-terminus. **a-d**) Degradation of trace labeled Keap1- (**a,c**) or Rsp5- (**b,d**) ubiquitinated Neh2Dual-Barnase-DHFR-$His_6$ or Neh2Dual-DHFR-Barnase-$His_6$ by 100 nM wild-type (**a,b**) or Rpn13-pru (**c,d**) proteasome in the presence or absence of 100 μM MTX (2 hours). Fragments containing either DHFR plus a small tail or DHFR-Barnase plus a small tail were formed in the presence of proteasome and MTX from the Neh2Dual-Barnase-DHFR and Neh2Dual-DHFR-Barnase constructs respectively, and were identified relative to size standard control constructs (Barnase-DHFR and DHFR). Fragments formed in the presence of MTX were then pulled down with NiNTA under denaturing conditions to verify the presence of an intact C-terminus, and thus degradation from the N-terminus. **e**) Ubiquitination of MBP-Neh2Dual-Barnase-Cy5-DHFR or Neh2Dual-Barnase-Cy5-DHFR, as detected by Cy5 fluorescence. **f**) Degradation of 20 nM ubiquitinated substrate by 100 nM proteasome in the presence of 500 μM NADPH (1 hour). Degradation of MBP-capped substrate is reduced (from ~60% to ~30%), presumably because proteasome must now initiate degradation internally. Both substrates give rise to fragments containing Cy5-labeled DHFR. * marks a nonspecific product found in the ubiquitination reaction. **g, h**) Degradation of 20 nM Rsp5- (**g**) or Keap1- (**h**) ubiquitinated substrates by mutant proteasome as in panel f (2 hour degradation assay). All mutants give rise to the same C-terminal DHFR containing fragment with or without the MBP cap at the N-terminus, indicating all are degrading from the N-terminus. Rpn13-pru consistently gives a slightly larger fragment.

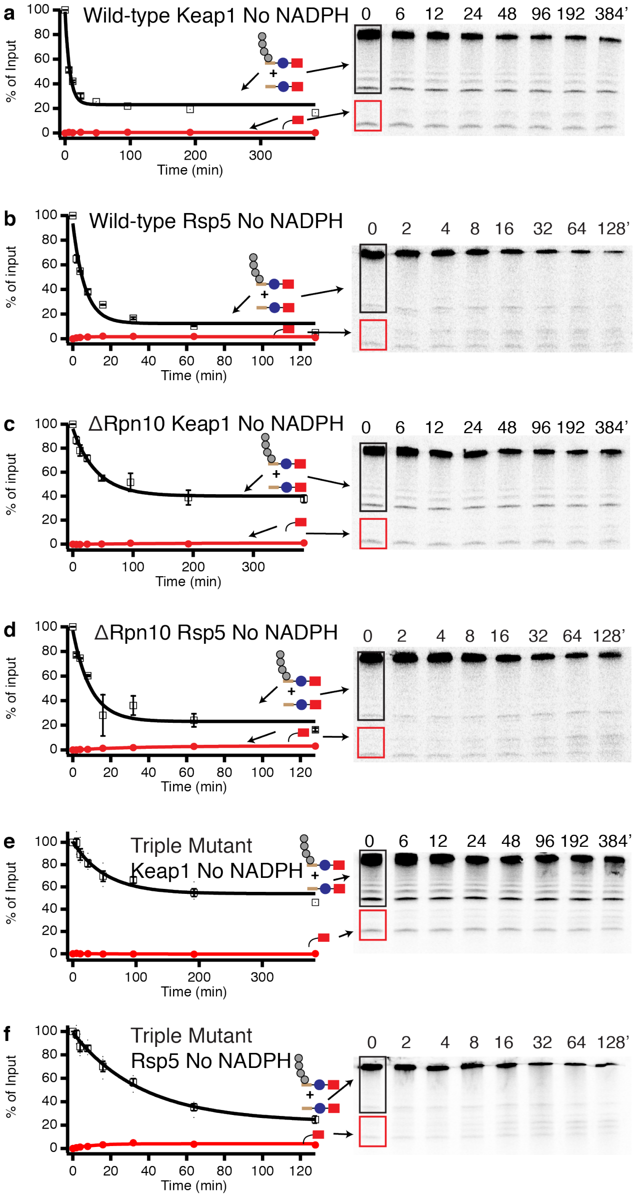


**Supplementary Figure S2**. Proteasome mutants can degrade weakly folded proteins. Degradation of trace radiolabeled Keap1- or Rsp5-ubiquitinated Neh2Dual-BarnaseL89G-DHFR substrate by 100 nM wild-type (**a**,**b**), ΔRpn10 (**c**,**d**), or triple-mutant ubiquitin receptor (**e**,**f**) proteasome (Rpn1ΔT1/Rpn10ΔUIM/Rpn13-pru). With a substrate containing a destabilized barnase domain and in the absence of NADPH, degradation occurs without or with minimal fragment formation (amplitude for fragment formation with Rsp5-ubiquitinated substrate with wild-type is 1.7 ± 0.4%, with ΔRpn10 is 3.3 ± 0.3%, and with the triple-mutant is 4.0 ± 0.5%; no fragment was observed with Keap1-ubiquitinated substrates for any of the proteasomes). The amounts of full-length protein (open squares) and DHFR fragment (red circles) are shown as a percentage of the full-length substrate presented to the proteasome at the beginning of the reaction. Dots are results from individual experiments, and error bars represent the SEM of 4 experiments. Curves are global fits to single exponentials.

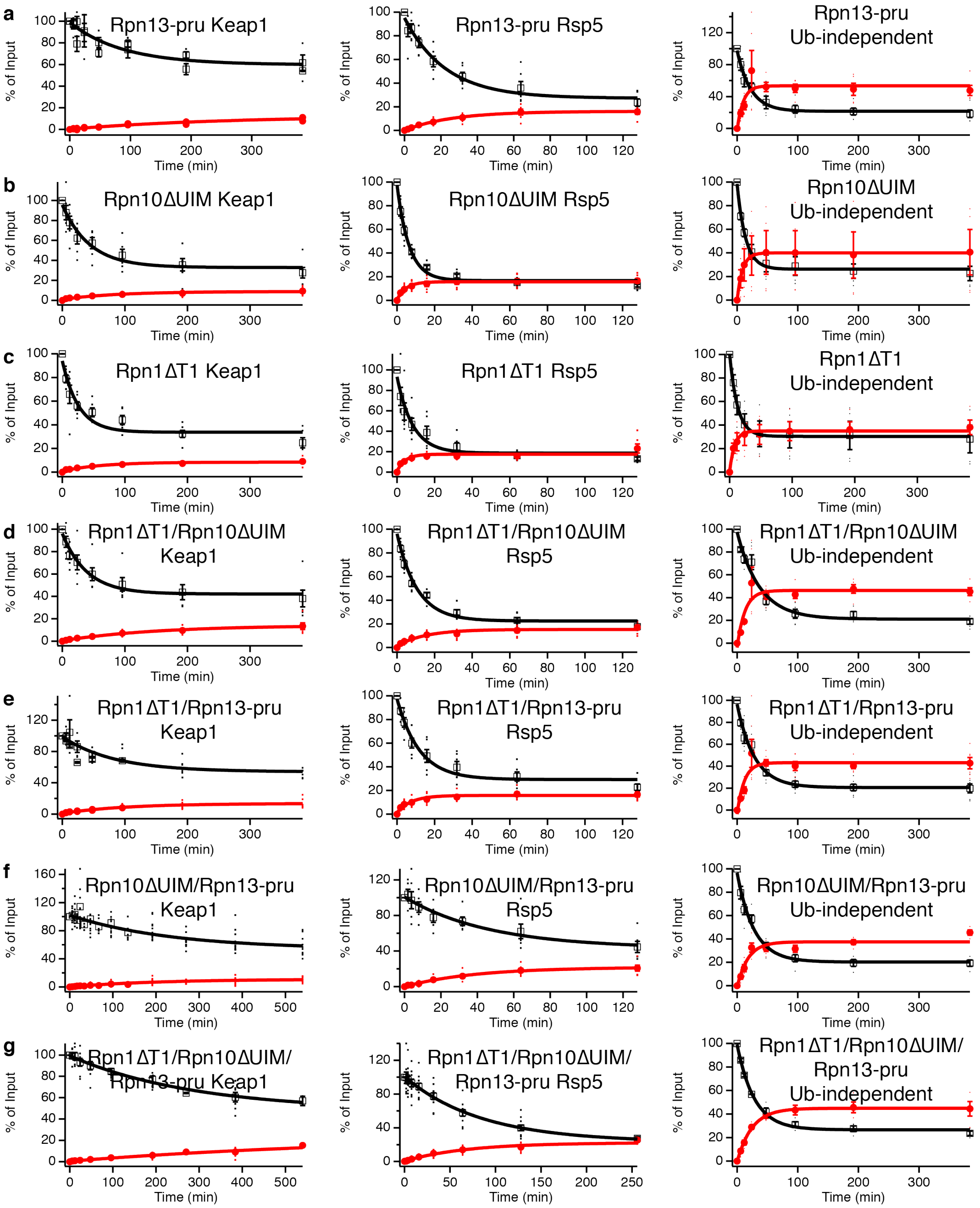


**Supplementary Figure S3**. Degradation assays for mutant proteasomes. Degradation of trace radiolabeled Keap1-, Rsp5-ubiquitinated, or ubiquitin-independent substrate by 100 nM Rpn13-pru (**a**), Rpn10ΔUIM (**b**), Rpn1ΔT1 (**c**), Rpn1ΔT1/Rpn10ΔUIM (**d**), Rpn1ΔT1/Rpn13-pru (**e**), Rpn10ΔUIM/Rpn13-pru (**f**), or Rpn1ΔT1/Rpn10ΔUIM/Rpn13-pru (**g**) proteasome. The amounts of full-length protein (open squares) and DHFR fragment (red circles) are shown as a percentage of the full-length substrate presented to the proteasome at the beginning of the reaction. Dots are results from individual experiments, and error bars represent the SEM of 4-15 experiments. Curves are global fits to single exponentials.

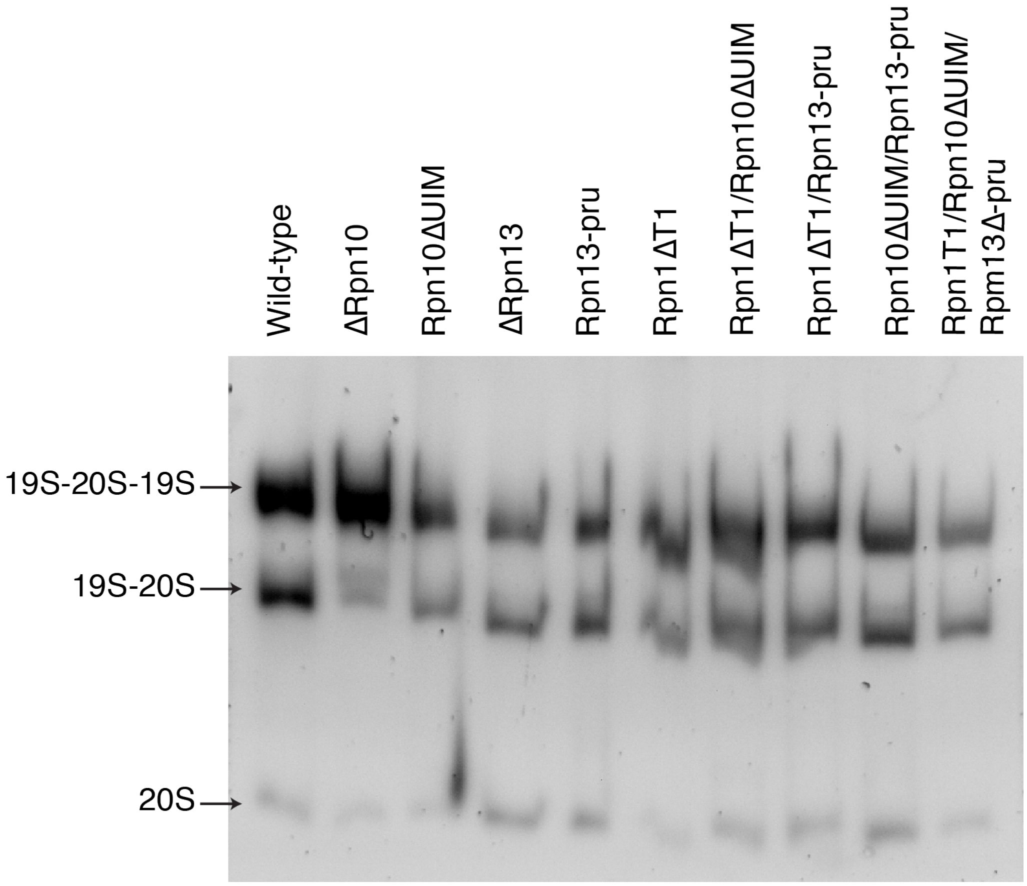


**Supplementary Figure S4**. Native gel analysis of proteasome preps. ~6 μg of each proteasome prep was run on a 3.5% native gel, followed by visualization in the presence of 50 μM Suc-LLVY-AMC and 0.02% SDS.

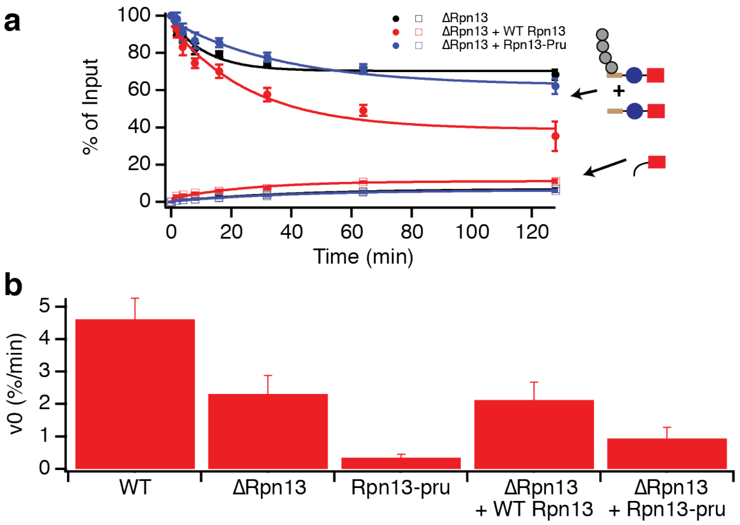


**Supplementary Figure S5**. Rpn13-pru is a more severe mutation than ΔRpn13. **a)** Degradation of trace radiolabeled Keap1- ubiquitinated Neh2Dual-BarnaseL89G-3Cpro-DHFRδK-His by 100 nM wild-type proteasome ± 250 nM His10-Rpn13 (WT or pru mutant). Adding WT Rpn13 increases the extent of degradation (although not the initial rate of degradation; see below), while adding Rpn13-pru slows degradation. **b)** Initial rates of degradation (calculated from amplitude and observed rate constant from **a** or from Figure 6 and 7) for add-back experiment compared to WT and Rpn13-pru proteasome.

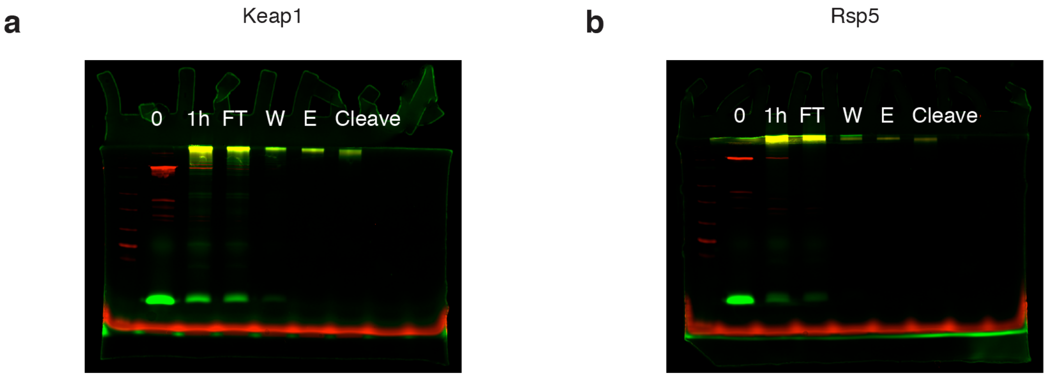


**Supplementary Figure S6**. Estimation of number of ubiquitins per substrate. An MBP-3C$_{site}$-Barnase-Cys$^{Cy5}$-DHFR-His construct (red) was ubiquitinated with either **a**) Keap1 or **b**) Rsp5 for 1 hour in the presence of Cy3-labeled ubiquitin (green), bound to amylose resin, washed, eluted with maltose, and cleaved with HRV 3C protease to remove the MBP domain. The intensities of Cy3 and Cy5 in the final purified ubiquitinated substrate was compared to the intensities before ubiquitination or purification (and the known initial concentrations of substrate and ubiquitin) to determine the number of ubiquitins per substrate by imaging on a Typhoon FLA9500. Based on replicate experiments and comparisons to both the 0 and 1 hour timepoints, we estimate 30 ± 4 ubiquitins/Keap1 substrate and 22 ± 10 ubiquitins/Rsp5 substrate (the lower yield of the Rsp5 substrate makes estimation more difficult, but manual counting of individual ubiquitin bands at early time points using both fluorescent and radiolabeled substrates suggests that both Rsp5- and Keap1-ubiquitinated substrates carry in excess of 10 ubiquitins).

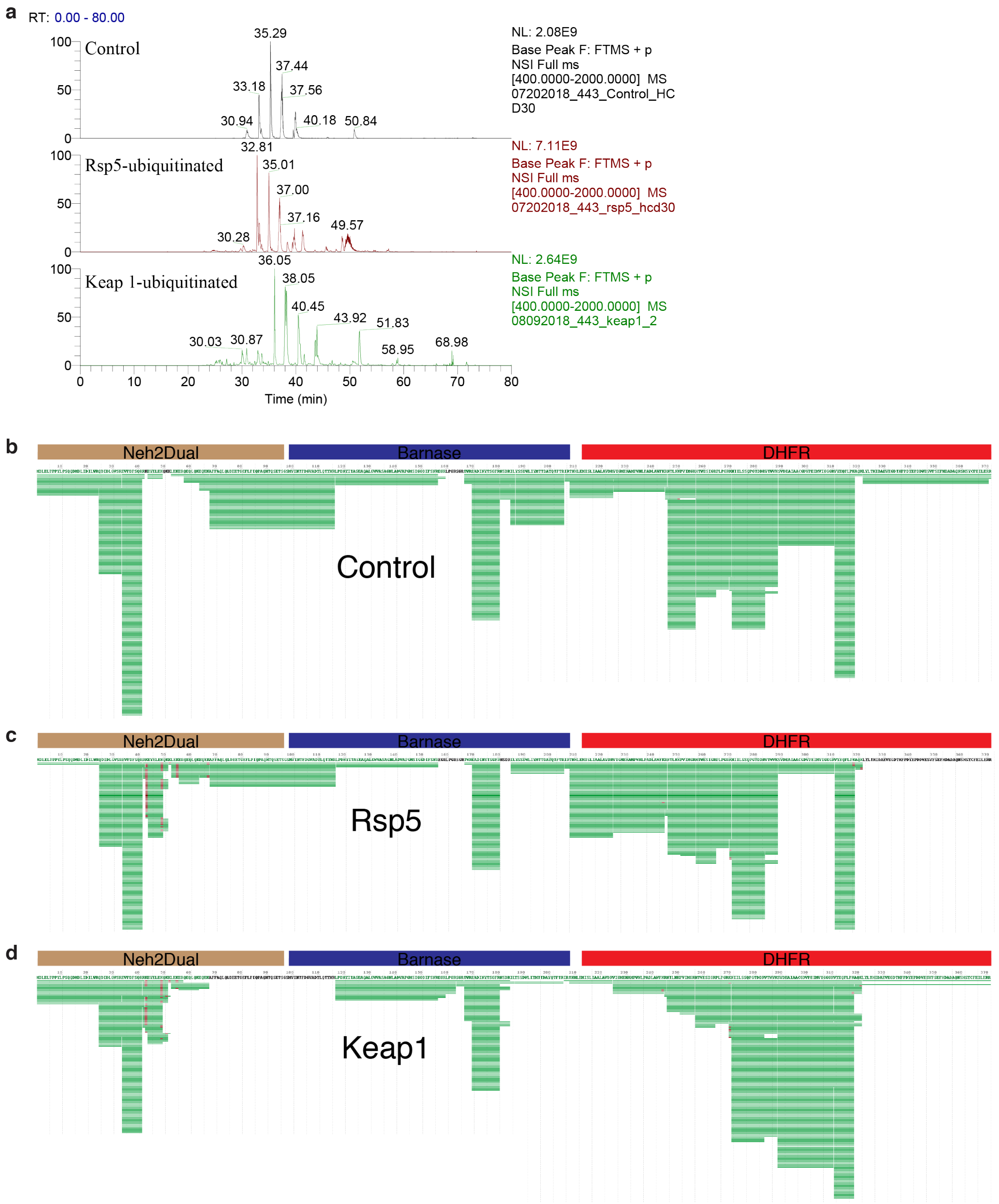


**Supplementary Figure S7**. Bottom-up mass spectrometry of Neh2Dual-Barnase-DHFR identifies ubiquitination sites. **a**) Representative LC traces obtained for control (non-ubiquitinated) and ubiquitinated samples after tryptic digestion. **b-d**) Peptide coverage maps for **b**) control, **c**) Rsp5-ubiquitinated and **d**) Keap1-ubiquitinated samples. Green lines are peptides, red marks are sites of ubiquitination.

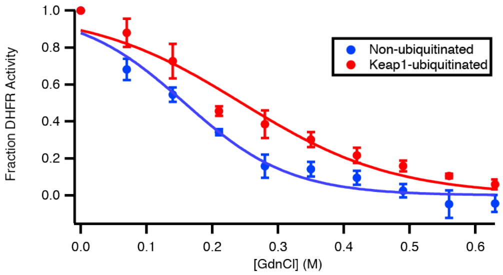


**Supplementary Figure S8.** Ubiquitination does not destabilize DHFR. Non- or Keap1-ubiquitinated Neh2Dual-Barnase-Cys-DHFR was assayed for activity in the presence of different concentrations of GdnCl. Data was fit to a two-state model:

$$\text{Fraction DHFR Activity} = \frac{e^{\left(\frac{\Delta G_D - m*x}{RT}\right)}}{1+e^{\left(\frac{\Delta G_D - m*x}{RT}\right)}}.$$

$\Delta G_D$ = 1.2 ± 0.2 kcal/mol for the non-ubiquitinated substrate and 1.3 ± 0.1 kcal $mol^{-1}$ for the Keap1-ubiquitinated substrate, m = 7.4 ± 0.8 and 5.1 ± 0.5 kcal $mol^{-1}$ $M^{-1}$, and $[GdnCl]_{1/2}$ = 0.16 ± 0.03 and 0.25 ± 0.04 M respectively.

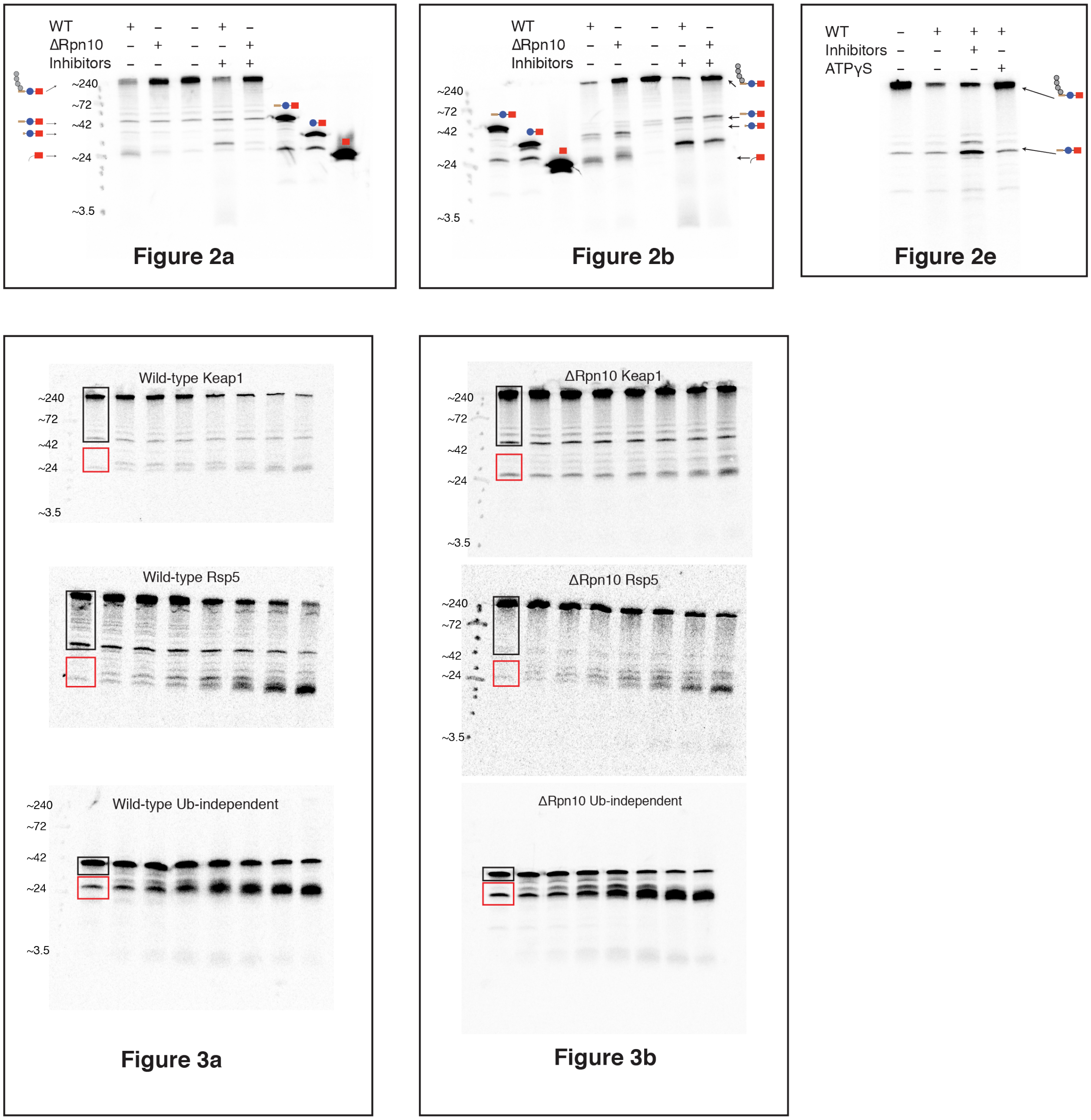


**Supplementary Figure S9**. Uncropped gels from Figures 2 and 3.

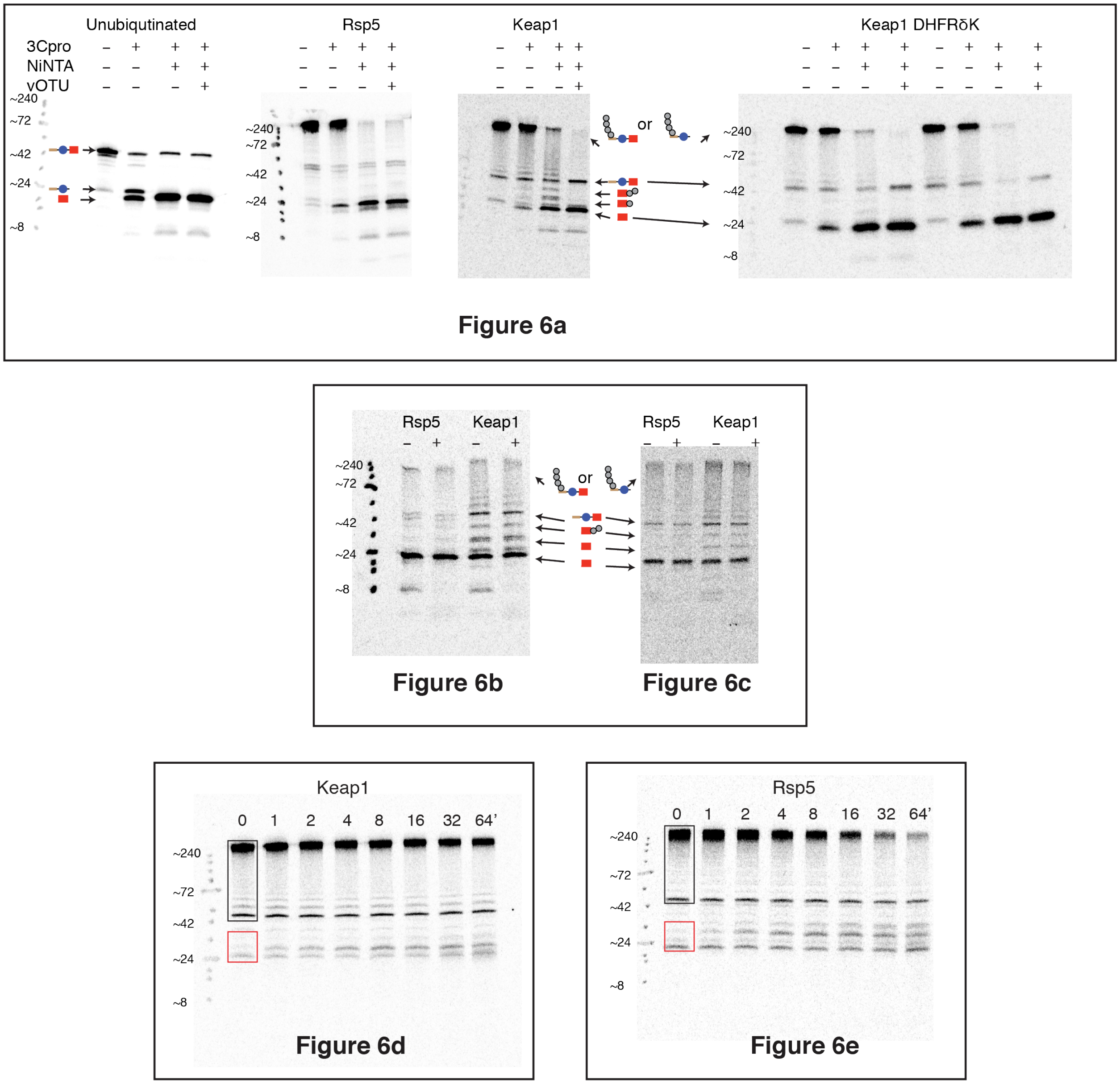


**Supplementary Figure S10.** Uncropped gels from Figure 6. For the DHFRδK gel in Figure 6a, the 4 lanes on the right are the bands shown in Figure 6a while the 4 lanes on the left are from a replicate experiment.

**Supplementary Table S1**. Summary of observed rate constants and unfolding abilities of wild-type and mutant proteasomes with Neh2Dual-BarnaseL89G-DHFR-His substrate. Errors are standard deviations propagated from global fitting.

| | Keap1-Ub | | Rsp5-Ub | | Ub-independent | |
|---|---|---|---|---|---|---|
| Mutant | $k_{obs}$ ($min^{-1}$) | U | $k_{obs}$ ($min^{-1}$) | U | $k_{obs}$ ($min^{-1}$) | U |
| WT | 0.051 ± 0.006 | 7.9 ± 0.8 | 0.14 ± 0.01 | 5.1 ± 0.6 | 0.033 ± 0.004 | 0.7 ± 0.2 |
| ΔRpn10 | 0.012 ± 0.007 | 4 ± 2 | 0.03 ± 0.01 | 2.5 ± 0.6 | 0.024 ± 0.006 | 0.5 ± 0.3 |
| ΔRpn13 | 0.020 ± 0.003 | 5.4 ± 0.6 | 0.05 ± 0.01 | 3.6 ± 0.5 | 0.027 ± 0.003 | 0.7 ± 0.3 |
| Rpn1ΔT1 | 0.038 ± 0.008 | 6.9 ± 0.9 | 0.12 ± 0.02 | 3.5 ± 0.6 | 0.08 ± 0.02 | 1.1 ± 0.5 |
| Rpn10ΔUIM | 0.023 ± 0.006 | 7 ± 1 | 0.16 ± 0.01 | 4.4 ± 0.6 | 0.07 ± 0.01 | 0.8 ± 0.9 |
| Rpn13-pru | 0.011 ± 0.004 | 2.2 ± 0.6 | 0.048 ± 0.007 | 2.7 ± 0.3 | 0.045 ± 0.008 | 0.4 ± 0.3 |
| Rpn1ΔT1/ Rpn10ΔUIM | 0.025 ± 0.007 | 2.9 ± 0.7 | 0.094 ± 0.009 | 4.3 ± 0.5 | 0.027 ± 0.004 | 0.5 ± 0.2 |
| Rpn1ΔT1/ Rpn13-pru | 0.012 ± 0.004 | 2.5 ± 0.6 | 0.08 ± 0.01 | 3.7 ± 0.6 | 0.035 ± 0.04 | 0.6 ± 0.2 |
| Rpn10ΔUIM/ Rpn13-pru | 0.004 ± 0.001 | 3.5 ± 0.8 | 0.02 ± 0.01 | 1.7 ± 0.5 | 0.036 ± 0.004 | 1.0 ± 0.2 |
| Rpn10ΔUIM/ Rpn13-pru* | 0.007 ± 0.004 | 4 ± 1 | 0.05 ± 0.01 | 2.2 ± 0.2 | ND | ND |
| Rpn1ΔT1/ Rpn10ΔUIM/ Rpn13-pru | 0.003 ± 0.001 | 1 ± 1 | 0.012 ± 0.003 | 2.6 ± 0.4 | 0.035 ± 0.002 | 0.6 ± 0.1 |

*Additionally purified via superose 6 gel filtration; data from 2 trials.

**Supplementary Table S2**. Number of modified and unmodified peptides containing lysine residues identified for Rsp5- and Keap1-ubiquitinated Neh2Dual-Barnase-DHFR substrate. (Only peptides with PEP2D score lower than 0.001 were considered).

| **Rsp5** | **No. of modified peptides** | **No. of unmodified peptides** | **Domain** |
|---|---|---|---|
| K43 | 32 | 2 | Neh2Dual |
| K49 | 19 | 27 | Neh2Dual |
| K52 | 1 | 0 | Neh2Dual |
| K55 | 5 | 0 | Neh2Dual |
| K67 | 4 | 5 | Neh2Dual |
| K244 | 1 | 52 | DHFR |
| K270 | 2 | 11 | DHFR |
| K318 | 3 | 119 | DHFR |
| **Keap1** | **No. of modified peptides** | **No. of unmodified peptides** | **Domain** |
| K43 | 24 | 9 | Neh2Dual |
| K49 | 13 | 28 | Neh2Dual |
| K244 | 3 | 11 | DHFR |
| K270 | 5 | 27 | DHFR |
| K318 | 2 | 95 | DHFR |

**Supplementary Table S3**. Summary of observed rate constants and unfolding abilities of wild-type and mutant proteasomes with Neh2Dual-BarnaseL89G-3CPro-DHFRδK-His substrate. Errors are standard deviations propagated from global fits.

| | Keap1-Ub | | Rsp5-Ub | |
|---|---|---|---|---|
| Mutant | $k_{obs}$ ($min^{-1}$) | U | $k_{obs}$ ($min^{-1}$) | U |
| WT | 0.09 ± 0.02 | 3.2 ± 0.4 | 0.21 ± 0.02 | 2.8 ± 0.2 |
| Rpn13-pru | 0.008 ± 0.002 | 1.1 ± 0.3 | 0.026 ± 0.004 | 1.6 ± 0.2 |
| Rpn1ΔT1/ Rpn10ΔUIM | 0.010 ± 0.004 | 0.4 ± 0.2 | 0.049 ± 0.004 | 2.4 ± 0.2 |
| Rpn10ΔUIM/ Rpn13-pru | < 0.01 | ND | 0.035 ± 0.006 | 0.9 ± 0.2 |

**Supplementary Table S4**. Yeast strains. All strains derived from YYS40[58].

| **Strain** | **Name** | **Genotype** |
|---|---|---|
| YYS40 | Wild-type | MATa RPN11-3FLAG::HIS3 |
| yNDN1 | ΔRpn10 | MATa RPN11-3FLAG::HIS3ΔRpn10::NatMX |
| yNDN2 | ΔRpn13 | MATa RPN11-3FLAG::HIS3ΔRpn13::NatMX |
| yDAK36 | Rpn1ΔT1 | MATa RPN11-3FLAG::HIS3 ΔRpn1::NatMX Rpn1ΔT1::LEU2 (cen plasmid) |
| yDAK34 | Rpn10ΔUIM | MATa RPN11-3FLAG::HIS3 ΔRpn10::NatMX Rpn10ΔUIM::LEU2 (cen plasmid) |
| yMDC3 | Rpn13-pru | MATa RPN11-3FLAG::HIS3 ΔRpn13::NatMX Rpn13-pru::URA3 (cen plasmid) |
| yDAK39 | Rpn1ΔT1/Rpn10ΔUIM | MATa RPN11-3FLAG::HIS3 ΔRpn1::KanMX ΔRpn10::NatMX Rpn1ΔT1::LEU2 (cen plasmid) Rpn10ΔUIM::URA3 (cen plasmid) |
| yDAK44 | Rpn1ΔT1/Rpn13-pru | MATa RPN11-3FLAG::HIS3 ΔRpn1::KanMX ΔRpn13::NatMX Rpn1ΔT1::LEU2 (cen plasmid) Rpn13ΔUIM::URA3 (cen plasmid) |
| yDAK45 | Rpn10ΔUIM/ Rpn13-pru | MATa RPN11-3FLAG::HIS3 ΔRpn10::KanMX ΔRpn13::NatMX Rpn10ΔUIM::LEU2 (cen plasmid) Rpn13-pru::URA3 (cen plasmid) |
| yDAK47 | Rpn1ΔT1/Rpn10ΔUIM/ Rpn13-pru | MATa RPN11-3FLAG::HIS3 ΔRpn1::KanMX ΔRpn13::NatMX Rpn1ΔT1::LEU2 (cen plasmid) Rpn13ΔUIM::URA3 (cen plasmid) Rpn10ΔUIM |
